# Role of Uterine Artery Doppler Ultrasound in Predicting Pre-Eclampsia in High-Risk Women

**DOI:** 10.7759/cureus.16276

**Published:** 2021-07-09

**Authors:** Nadia Shahid, Mehar Masood, Zakia Bano, Urooj Naz, Syeda Fariha Hussain, Adnan Anwar, Atif A Hashmi

**Affiliations:** 1 Obstetrics and Gynaecology, Sindh Government Hospital Liaquatabad, Karachi, PAK; 2 Obstetrics and Gynaecology, Liaquat College of Medicine and Dentistry, Karachi, PAK; 3 Obstetrics and Gynaecology, Dow University of Health Sciences, Karachi, PAK; 4 Obstetrics and Gynaecology, Jinnah Medical and Dental College, Karachi, PAK; 5 Physiology, Hamdard College of Medicine and Dentistry, Karachi, PAK; 6 Pathology, Liaquat National Hospital and Medical College, Karachi, PAK

**Keywords:** pre-eclampsia, maternal mortality, doppler ultrasonography, uterine artery notching, resistance index

## Abstract

Background and objective

Pre-eclampsia (PE) is a major cause of maternal morbidity and mortality. The utility of Doppler ultrasonography (U/S) in predicting PE has not been extensively explored. This study aimed to determine the role of Doppler U/S in predicting PE among high-risk women.

Methodology

This was a retrospective observational study conducted at the Department of Obstetrics and Gynecology of Abbasi Shaheed Hospital in Karachi, over a period of one year, from January 2019 till December 2019. A total of 325 women were initially screened for risk factors for PE. Among them, 75 women were eventually found to have risk factors for PE and hence included in the study. Uterine artery Doppler U/S was performed to evaluate uterine artery’s flow velocity waveforms. They were then used to calculate the presence of diastolic notch and resistance index (RI). At each antenatal visit, the risk factors for PE such as BP, proteinuria, and signs and symptoms were noted. Women were labeled to have PE if they developed hypertension (BP >140/90) after 20 weeks of gestation in combination with proteinuria.

Results

Twenty women (28%) had a normal Doppler flow of the uterine arteries. In 54 (72%) women, a unilateral/bilateral RI >0.58 was observed, and 29 women (38.7%) had a bilateral Rl >0.58. Notching of the uterine artery was also observed in 42 (26.7%, unilateral/bilateral) and in 22 (29.3%) bilaterally. Among the 75 women, BP of 140/90 mmHg along with proteinuria was observed in 56 (76.7%) cases, which were hence diagnosed as PE. Based on the cutoff of Rl and notching of the uterine artery, the overall sensitivity, specificity, and positive and negative predictive values (PPV and NPV) of Doppler U/S in predicting PE were 71.4%, 26.3%, 23.8%, and 74.1%, respectively. As far as individual Doppler U/S indices were concerned, RI >0.58 (unilateral/bilateral) was found to be most sensitive (71%), while the presence of uterine artery notch (unilateral/bilateral) was most specific in predicting PE.

Conclusion

Abnormal Doppler U/S has good overall sensitivity in predicting PE. Among individual Doppler indices, notching of uterine arteries had a better specificity compared to high RI.

## Introduction

Pre-eclampsia (PE) is a condition involving multiple organ systems, which originates during early pregnancy and can lead to substantial maternal mortality and morbidity. However, the pathophysiology of PE is still not clear as to how it involves both the fetal/placental as well as maternal factors [1]. The primary cause of PE is attributed to relatively under-perfused/hypoxic/ischemic placenta, probably due to the abnormal development of placental vasculature early in the duration of pregnancy. The estimated rate of PE is around 10%, and it is a major cause of iatrogenic pre-term births [2].

Like any other condition, the early diagnosis of PE during pregnancy is needed to plan appropriate treatment and the monitoring of management. Complications can be effectively contained if PE is diagnosed as early as possible [3]. Hypertension in pregnancy can affect up to 10% of expectant mothers. Substantial variations have been reported between developing and developing countries, owing to the differences in socioeconomic factors and data collection [4]. PE along with its complications plays a significant role in maternal as well as perinatal morbidity and mortality globally. With effective and timely management, the outcomes in women with PE can be significantly improved. This can be achieved by developing effective methods for predicting and preventing PE and its complications so that optimal prenatal care can be provided [5].

With the use of ultrasonography (U/S) for predicting/screening PE, it was observed that PE due to defective placentation causes an incomplete transformation of spiral arteries [6]. A lesion of placental villi and vascular histopathology is four to seven times more commonly seen in PE as compared to non-PE pregnancies [7]. They are linked to an increase in resistance to the flow of the uterine artery. In measuring the impedance (resistance) to the flow of uterine arteries through Doppler U/S, assessing and quantifying incomplete spiral arteries' transformation can be performed [8,9].

The objective of this study was to determine the role of Doppler U/S in predicting PE among high-risk women.

## Materials and methods

This was a retrospective observational study conducted at the Department of Obstetrics and Gynecology of Abbasi Shaheed Hospital in Karachi, over a period of one year, from January 2019 till December 2019. A total of 325 women were initially screened for risk factors for PE. The risk factors for PE included a previous history or a family history of PE, diabetes mellitus, age above 30 years, history of polycystic ovarian syndrome, urinary tract infection, or a previous history of pre-term birth. Pregnant mothers having no high-risk factors for PE and those who were not willing to participate in the study were excluded. Women with uncontrolled hypertension before 20 weeks of gestation or before pregnancy were also excluded.

After obtaining informed consent from the patients, their data were collected. Based on the inclusion and exclusion criteria, a total of 75 pregnant women were included in the study. A detailed history of the mothers including age, weight, height, body mass index (BMI), and any previous medial or obstetrical history was noted. Gestational age was calculated based on the last menstrual period or from an earlier scan. Both general physical and systemic examinations were carried out in detail. Laboratory investigations included complete blood counts, blood grouping, detailed urine report, random blood sugar (RBS), and two clean catch of mid-stream urine were collected more than four hours apart on a reagent strip and those having >2 proteinuria were recorded. Women were labeled to have PE if they developed hypertension (BP >140/90) after 20 weeks of gestation coupled with proteinuria. The machine used for Doppler U/S was a Toshiba Nemio 30 with color Doppler (Canon Medical Systems Corporation, Ōtawara, Japan). For Doppler U/S, the mothers were positioned in a semi-recumbent way with a transducer laced on the left and right lower quadrants of the maternal abdominal wall, which enabled the visualization of the external iliac artery and identification of the uterine artery’s flow velocity waveforms. They were then used to calculate the presence of diastolic notch and resistance index (RI). At each antenatal visit, the risk factors for PE such as BP, proteinuria, and signs and symptoms were noted. All data were recorded on a pre-designed proforma.

Data analysis

For data analysis, SPSS Statistics Version 26.0 (IBM Inc., Armonk, NY) was used. Using cross-tabulation, the sensitivity, specificity, as well as positive and negative predictive values (PPV and NPV) were reported for Doppler U/S.

## Results

Among the total 75 pregnant mothers included in the study, the mean maternal age was 27.65 ±4.77 years, while the mean gestational age was 23.88 ±1.82 weeks. Regarding risk factors of PE, a previous history of PE was the most common factor observed (52, 69.3%) among the cases, followed by a family history of PE (49, 65.3%), as shown in Table 1.

**Table 1:** Baseline demographics and risk factor of pre-eclampsia among patients included in the study (n=75) SD: standard deviation

Variables	Values
Maternal age, years, mean ±SD	27.65 ±4.77
Gestational age, weeks, mean ±SD	23.88 ±1.82
Previous history of pre-eclampsia, n (%)	52 (69%)
Family history of pre-eclampsia, n (%)	49 (65%)
Diabetes mellitus, n (%)	13 (17%)
Urinary tract infection, n (%)	09 (12%)
Polycystic ovarian syndrome, n (%)	04 (5%)
Renal disease, n (%)	03 (4%)

**Figure 1:**
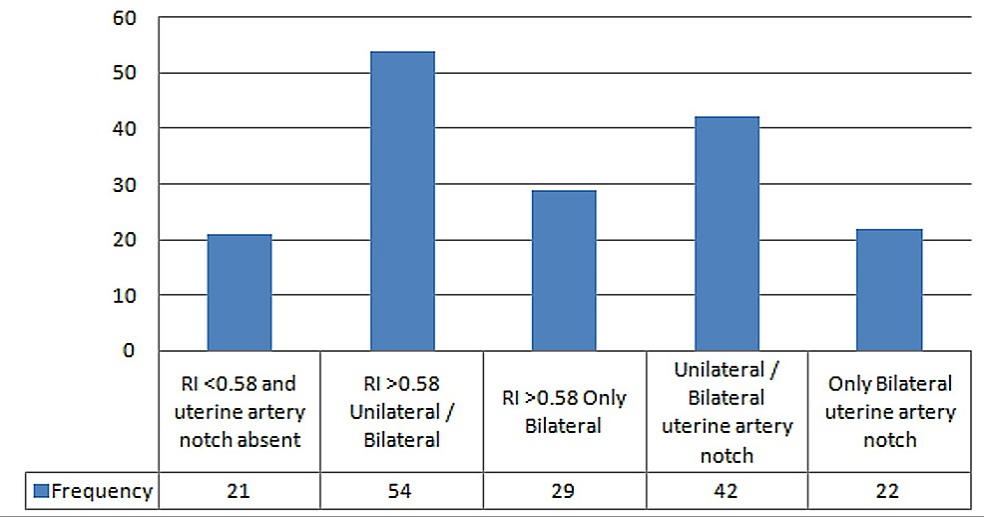
Graphical representation of Doppler ultrasound findings RI: resistance index

Twenty women (28%) had a normal Doppler flow of the uterine arteries. In 54 (72%) women, a unilateral/bilateral RI >0.58 was observed, and 29 women (38.7%) had a bilateral Rl >0.58. Notching of the uterine artery was also observed in 42 (26.7%, unilateral/bilateral) and in 22 (29.3%) bilaterally. The Doppler ultrasound findings of uterine arteries are shown in Figure 1.

**Figure 2:**
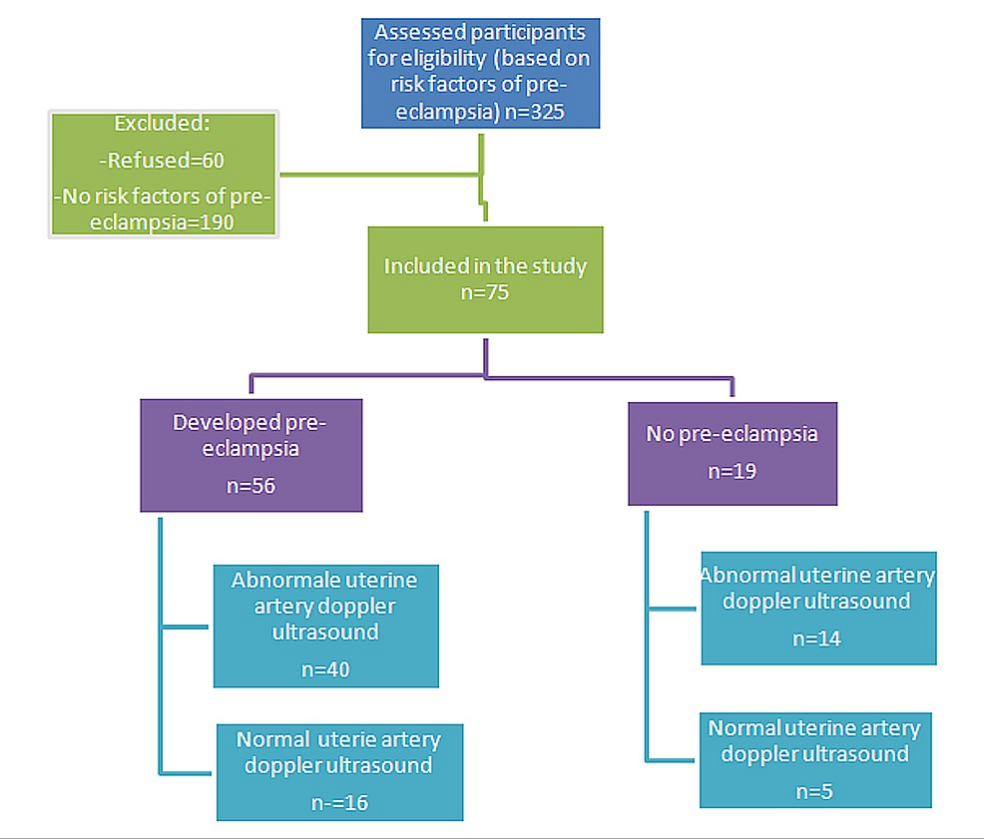
Patients' flow chart

Out of 75 women, BP of 140/90 mmHg along with proteinuria was observed in 56 (76.7%) cases, and they were diagnosed as PE patients. Out of these 56 patients, 40 cases had abnormal uterine artery Doppler U/S. On the other hand, among 19 women who did not develop PE, abnormal uterine artery Doppler U/S was noted in five cases, as shown in Figure 2.

Based on the cutoff of Rl and notching of the uterine artery, the sensitivity, specificity, PPV, and NPV of Doppler U/S in predicting PE were 71.4%, 26.3%, 23.8%, and 74.1%, respectively (Table 2).

**Table 2:** Overall sensitivity, specificity, PPV, and NPV of an abnormal Doppler ultrasound in predicting pre-eclampsia RI: resistance index; PPV: positive predictive value; NPV: negative predictive value

Doppler ultrasound findings		Pre-eclampsia (blood pressure >140/90 mmHg and proteinuria)		Total	Sensitivity	Specificity	PPV	NPV
		Yes	No					
Abnormal	(Uni/bilateral RI >0.58 and uni/bilateral notch)	40	14	54 (72%)	71.4%	26.3%	23.8%	74.1%
Normal	(RI ≤0.58 and absent uterine artery notch)	16	5	21 (28%)				
Total		56 (76.7%)	19 (25.3%)	75				

As far as individual Doppler U/S indices were concerned, RI >0.58 (unilateral/bilateral) was most sensitive (71%), while the presence of uterine artery notch (unilateral/bilateral) was most specific in predicting PE. The sensitivity of bilateral RI >0.58 was low (41%) compared to unilateral RI >0.58; however, the presence of bilateral RI >0.58 was more specific. Similarly, the presence of bilateral notching of the uterine artery was more specific in predicting PE (79%) compared to unilateral notching (Table 3).

**Table 3:** Sensitivity, specificity, PPV, and NPV of Doppler indices in predicting pre-eclampsia RI: resistance index; TP: true positive; FP: false positive; FN: false negative; TN: true negative; PPV: positive predictive value; NPV: negative predictive value

Doppler indices	TP	FP	FN	TN	Sensitivity	Specificity	PPV	NPV
Uni/bilateral RI >0.58 (any RI >0.58)	40	14	16	5	71%	26%	74%	24%
Bilateral RI >0.58 (both RI >0.58)	23	7	33	12	41%	63%	77%	27%
Uni/bilateral notch (any notch)	40	2	16	17	71%	89%	95%	52%
Bilateral notch (both notch)	18	4	38	15	32%	79%	84%	28%

## Discussion

Different studies have reported varying frequencies of PE and related findings on Doppler U/S [10]. A study has observed high impedance flow in the uterine artery in about 40% of pregnant mothers, who subsequently developed PE [11]. It has also been reported that after a positive ultrasound scan on notching or impedance to flow of uterine arteries, the likelihood for PE increases by about two folds. Since PE is regarded as the most common cause of maternal mortality and morbidity, an accurate identification or prediction of PE, especially in high-risk mothers, is vital for providing timely intervention, which may prove crucial in improving maternal as well as fetal outcomes [12].

Another study has reported a PE frequency of 11.5% in which RI was >0.58 and the mothers were in-between 18-24 weeks of gestation [13]. In yet another study, 6% of mothers were found to have PE in between 20-24 weeks of gestation and an RI >0.58 [14]. However, in our study, a much higher rate of 76.7% was observed in terms of PE in between 20-26 weeks of gestation. Similar to our study, one research has reported a PE rate of 55% in mothers who had uterine artery notch bilaterally at 24 weeks of gestation, which increased to 81% at the time of delivery, with all such mothers giving birth prior to 35 weeks of gestation [15].

In this study, for the Doppler index of unilateral/bilateral RI >0.58, the sensitivity, specificity, PPV, and NPV were 71%, 26%, 74%, and 24%, respectively; for bilateral RI >0.58, those were 41%, 63%, 77%, and 27%, respectively. For unilateral/bilateral uterine artery notch, these parameters were 71%, 89%, 95%, and 52%, and for bilateral notch, these were 32%, 79%, 84%, and 28%, respectively. Compared to our study, another study has observed that the PPV in the notch of the uterine artery was 25%, with PE frequency reported in 18% while the sensitivity, specificity, PPV, and NPV in RI >0.58 were 41%, 96%, 70%, and 88%, respectively. Likewise, the values for bilateral uterine artery notching were 62%, 89%, 47%, and 94%, respectively. Various studies have reported PPV ranging from 35 to 60% and NPV ranging from 70 to 95% on the basis of PE diagnosed or predicted using Doppler U/S rather than assessing PE risk clinically [16].

One study has reported the prevalence of PE to be 58% among high-risk mothers who developed hypertension [17]. In yet another research, the abnormal Doppler ultrasound finding were reported in 11.3% of mothers with sensitivity, specificity, PPV, and NPV for PE at 36%, 90%, 11%, and 98%, respectively [18].

Similar to our study, some other studies have also observed that previous history of PE, smoking, nulliparity, first-trimester BMI >30 kg/m^2^, and a positive family history of PE are all risk factors for PE. In addition, with the introduction of Doppler U/S as a screening test for predicting PE, it has now become a test of prime importance [19].

Limitations of this study include the small sample size, retrospective study design, and the fact that data were drawn from a single institution only. Therefore, we recommend large-scale prospective studies to better understand the role of Doppler U/S in predicting PE.

## Conclusions

Based on our findings, using Doppler U/S for predicting PE by determining the notching of the uterine artery and its RI was successful in terms of abnormal uterine artery notching or high RI (>0.58), and it led to predicting PE in a majority of the patients. However, as our study was retrospective in design with a limited sample size, more large-scale prospective studies are recommended to validate these observations among our population.
